# Black Phosphorus Nanosheets‐Loaded Mussel‐Inspired Hydrogel with Wet Adhesion, Photothermal Antimicrobial, and In Situ Remineralization Capabilities for Caries Prevention

**DOI:** 10.1002/advs.202409155

**Published:** 2024-10-11

**Authors:** Ying Ran, Jiayi Shi, Yiqin Ding, Lujian Li, Dandan Lu, Youyun Zeng, Dongchao Qiu, Jie Yu, Xiaojun Cai, Yihuai Pan

**Affiliations:** ^1^ School and Hospital of Stomatology Wenzhou Medical University Wenzhou 325027 China; ^2^ Department of Endodontics School and Hospital of Stomatology Wenzhou Medical University Wenzhou 325027 China

**Keywords:** black phosphorus nanosheets, caries prevention, in situ remineralization, photothermal antimicrobial therapy, wet adhesion

## Abstract

The main features of early caries are the massive colonization of cariogenic bacteria and demineralization of tooth enamel by the acids that they produce. Owing to the lack of effective treatments, the development of anticaries therapeutics with both antimicrobial and remineralizing properties is urgently required. Black phosphorus nanosheets (BPNs) are ideal therapeutics for the treatment of early caries because they can mediate photothermal antibacterial activity and subsequently promote remineralization by generating PO_4_^3−^. However, the dynamic and wet environment of the oral cavity prevents the long‐term adhesion of BPNs to the tooth surface. In this study, using catechol‐modified chitosan and PLGA‐PEG‐PLGA as raw materials, a mussel‐inspired versatile hydrogel, BP@CP5, is presented that can be used to physically load BPNs. BP@CP5 has exceptional injectability and can firmly adhere to tooth surfaces for up to 24 h. Upon irradiation, BP@CP5 can quickly eliminate ≈99% of *Streptococcus mutans* and *Streptococcus sanguinis*; furthermore, the PO_4_^3−^ generated via degradation also promotes rapid remineralization of enamel slabs. Importantly, the vivo rodent caries modeling results further confirm the excellent caries‐prevention properties of BP@CP5. This study demonstrates that BP@CP5 is a promising anticaries material for caries management.

## Graphical abstract

A multifunctional hydrogel dressing (BP@CP5) for caries prevention is developed by physically loading of black phosphorus nanosheets (BPNs) within catechol‐modified chitosan and PLGA‐PEG‐PLGA hydrogels. BP@CP5 can firmly adhere to the tooth surface, ensuring the retention of BPNs. Benefiting from the photothermal activity and biodegradation products of BPNs, BP@CP5 is demonstrated to yield bactericidal and remineralization‐promoting effects with high efficiency.

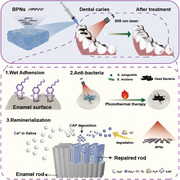

## 1 Introduction

Dental caries is a prevalent chronic disease affecting ≈2.44 billion people globally.^[^ ^1^ ^]^ It is a significant public health concern owing to its extensive impact, high prevalence, and often overlooked early symptoms.^[^ ^2^ ^]^ The initial stage of caries development, known as early caries, is characterized by structural alterations in the enamel, typically presenting as shallow cavities or white spot lesions.^[^ ^3^ ^]^ Cariogenic bacteria continue to produce organic acids on the tooth surface without sufficient preventive and therapeutic action, which leads to the demineralization of dental hard tissues and eventually causes tooth disintegration and cavities.^[^ ^4^ ^]^ Once dental caries progresses to this stage, the affected tooth tissue must be extracted, repaired, and replaced with an artificial filling material. Although this technique can repair defects, artificial filling materials are neither aesthetic nor as effective as natural teeth, and complications can lead to various health and economic problems.^[^ ^5^ ^]^ Thus, effectively controlling early caries is crucial for maintaining oral health.

Effective control of early caries entails the eradication of these bacteria as the demineralized tooth surface is repaired to maintain tooth integrity.^[^ ^4^, ^6^ ^]^ Currently, antibiotics, such as chlorhexidine and triclosan, are widely used to eliminate cariogenic bacteria, whereas fluoride is used to prevent demineralization. However, long‐term use of chlorhexidine and triclosan can lead to tooth staining, multidrug resistance in oral bacteria, oral flora imbalance, and potential systemic health impacts.^[^ ^7^ ^]^ In addition, excessive fluoride accumulation from treatments, such as varnish and fluoride, can cause dental and skeletal fluorosis.^[^ ^1^, ^8^ ^]^ On the other hand, numerous innovative approaches to treating dental caries have been reported, encompassing Photodynamic Therapy (PDT), Cocrystal engineering, antimicrobial peptides, and nanotechnology‐based particles and so on. However, these methods often face challenges such as difficulty in material removal or limitations in their therapeutic scope.^[^ ^9^ ^]^ Thus, there is an urgent need to develop new therapeutics with antimicrobial and remineralization properties to control early caries effectively.

Photothermal therapy (PTT) is an emerging non‐invasive treatment that utilizes the elevated temperatures generated by photothermal agents (PTAs) to disrupt bacterial cells and biofilm structures.^[^ ^10^ ^]^ PTT is currently garnering attention as a potential alternative to conventional antibiotic treatments for infections owing to its unique spatiotemporal controllability and reduced risk of bacterial resistance.^[^ ^11^ ^]^ Black phosphorus nanosheets (BPNs) are emerging 2D nanomaterials with excellent photovoltaic properties, good biocompatibility, and strong photothermal effects.^[^ ^12^ ^]^ Among them, as PTAs, BPNs have been widely used in antimicrobial and antibiofilm research.^[^ ^13^ ^]^ Furthermore, BPNs also have excellent biodegradability and can degrade in the presence of oxygen and water, producing PO_4_^3−^ ions, which are essential for biomineralization in hard tissue such as teeth and bones.^[^ ^14^ ^]^ Thus, the combination of BPN‐mediated in situ remineralization and photothermal antimicrobials presents a new strategy for early caries control, potentially providing advantages over existing approaches.

Despite BPNs being ideal materials for early caries control, the dynamic and wet environment of the oral cavity prevents them from being retained on the tooth surface long enough to yield a significant therapeutic effect.^[^ ^15^ ^]^ However, the mussel‐inspired hydrogel provides a perfect solution for the long‐term retention of BPNs on carious tooth surfaces,^[^ ^16^ ^]^ primarily because of the 1) 3D network structure of the hydrogel that can be used to efficiently load BPNs and 2) wet adhesion properties provided by the mussel‐inspired chemistry, which allows the hydrogel to adhere to carious surfaces for a long period of time.^[^ ^15^, ^17^ ^]^

**Scheme 1:**
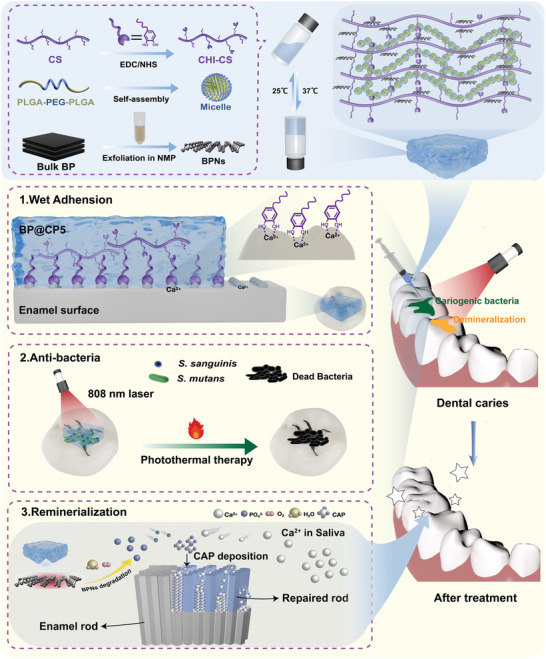
Schematic illustration of the preparation of the BP@CP5 hydrogel and its therapeutic mechanism to control early caries. 1) Wet adhesion properties. 2) BPNs‐mediated PTT against cariogenic bacteria. 3) Remineralization based on BPNs degradation.

Thus, we propose the construction of a multifunctional hydrogel dressing (BP@CP5) through the physical loading of BPNs within catechol‐modified chitosan (CHI‐CS) and PLGA‐PEG‐PLGA (PPP) hydrogels (**Scheme** **1**). Chitosan (CS), a cationic polysaccharide, exhibits certain bactericidal effects and tightly binds to negatively charged BPNs, enhancing their stability within the hydrogel.^[^ ^18^ ^]^ Catechol modification enhances the wet adhesion property of the hydrogel, and PPP imparts injectable and temperature‐sensitive characteristics to the hydrogel.^[^ ^19^ ^]^

Leveraging the advantages of BPNs and mussel‐inspired chemistry, we hypothesized that the BP@CP5 hydrogel will exhibit the following distinctive characteristics: 1) excellent wet adhesion, 2) BPN‐mediated PTT for efficient bactericidal action, and 3) BPNs degradation to promote in situ remineralization. We systematically evaluated the wet adhesion capacity, photothermal antimicrobial activity, and remineralization properties by conducting in vitro experiments. We also validated the ability of the proposed hydrogel to prevent dental caries by establishing and analyzing a rat caries model.

## 2 Results and Discussion

### 2.1 Synthesis and Characterization BP@CP5

As shown in Scheme 1, the construction of BP@CP5 was primarily based on utilizing CHI‐CS and PPP as carriers for the physical loading of the BPNs. To this end, we first prepared BPNs using liquid‐phase exfoliation,^[^ ^14a^ ^]^ and CHI‐CS synthesis by coupling catechol to CS via an EDC/NHS‐mediated coupling reaction.^[^ ^20^ ^]^

**Figure 1:**
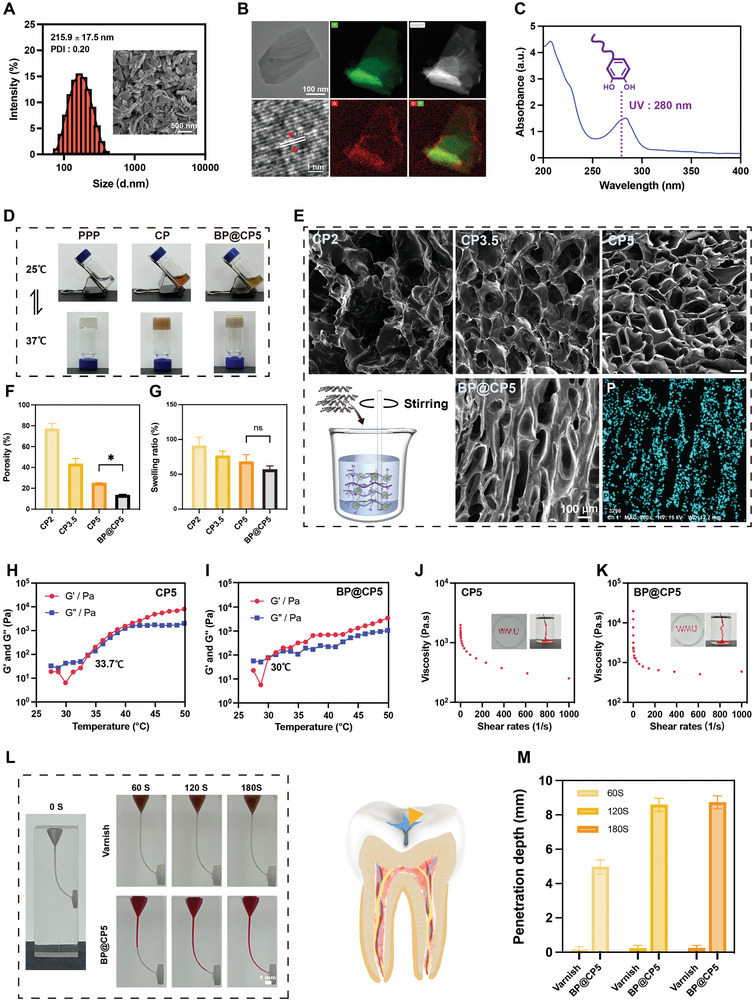
Physicochemical characterization of BP@CP5. A) Size distribution and SEM image of BPNs. B) TEM images and elemental mapping of BPNs. C) UV–vis absorption spectra of CHI‐CS in an aqueous solution. D) Images of various hydrogels with a sol–gel transition. E) SEM images of various freeze‐dried hydrogels and schematic diagram of CP‐loaded BPNs. Porosity F) and swelling G) proportions for various hydrogels. H, I) Temperature‐responsive storage (G’) and loss (G’’) moduli of various hydrogels as a function of temperature within the range of 25–50 °C. Shear viscosity and photographs (insert) showing the injectability of CP5 (J) and BP@CP5 (K). L) Illustration of the penetration capability of the varnish (control) and BP@CP5 (rhodamine B staining). M) Quantified results corresponding to (L), showing the change in penetration depth over time. In (F) and (G), the comparison is between BP@CP5 and CP5 (*n* = 3), * (*p* < 0.05), “ns” (Non‐significant).

The successful synthesis of the BPNs was confirmed by applying dynamic light scattering (DLS), scanning electron microscopy (SEM), and transmission electron microscopy (TEM), among other techniques. As illustrated in **Figure** **1**A, the BPNs had an average diameter of 215.9 ± 17.5 nm, with a polydispersity index of 0.20, indicating a homogeneous size distribution. The inset in Figure 1A shows an SEM image that confirms the homogeneous morphology of the BPNs. TEM analysis revealed the spacing of the lattice fringes of BPNs to be 0.22 nm, consistent with the structure of a black phosphorous monolayer.^[^ ^14b^ ^]^ Furthermore, the results of energy‐dispersive spectrometry (EDS) mapping (Figure 1B; Figure S1, Supporting Information) indicated that the BPNs primarily comprised oxygen (O) and phosphorus (P), which is consistent with literature reports.^[^ ^21^ ^]^ These results confirmed that the BPNs were successfully exfoliated into uniform nanosheets with the expected physical and chemical properties.

To verify the successful coupling of catechol and CS, we analyzed the product by applying UV–vis absorption spectroscopy and ^1^H NMR spectroscopy. As shown in Figure 1C, the UV–vis absorption spectrum included an absorption peak at 280 nm, which corresponded to the catechol bond absorption peak and confirmed the presence of catechol in CHI‐CS.^[^ ^17c^ ^]^ The ^1^H NMR spectrum included proton peaks near δ 6.8–7.0, which are characteristic of the benzene ring protons of catechol (Figure S2, Supporting Information). Furthermore, the grafting rate of catechol was calculated to be 11% based on the integral area of the proton peaks at the C‐2 position on the carbon ring of the CS main chain.^[^ ^22^ ^]^ These confirmed that catechol was successfully coupled to CS, resulting in the successful synthesis of CHI‐CS.

Following the successful preparation of the BPNs and CHI‐CS, we termed the hydrogel blended with CHI‐CS and PPP as CP and examined the gel‐forming abilities of PPP and CP. PPP is known for its excellent biodegradability and biocompatibility.^[^ ^23^ ^]^ In aqueous solutions, the polymer chains can self‐assemble into core–shell micelles, with hydrophobic PLGA constituting the core and hydrophilic PEG forming the shell.^[^ ^24^ ^]^ Upon elevating the temperature, we observed these micelles aggregate to form a gel, allowing PPP to be extensively used to prepare temperature‐sensitive hydrogels. Using the test‐tube inversion method, we visually inspected the temperature‐sensitive characteristics of the PPP and CP hydrogels.^[^ ^25^ ^]^ Both PPP and CP exhibited sol behavior under 25 °C and formed stable hydrogels at 37 °C (Figure 1D). We also discovered that the CP's average viscosity at 37 °C was 9249.0 Pa s, which was higher than that at 25 °C (3895.7 Pa s) (Figure S3, Supporting Information). This finding further highlights CP's temperature‐sensitive capability, as it exhibited a higher cross‐link density at 37 °C, leading to a corresponding increase in viscosity. Furthermore, the gelation period of the hydrogel was extended with the addition of CHI‐CS (Figure S4, Supporting Information), possibly because the PPP concentration was diluted.

Considering that the physicochemical and adhesion properties of the CP hydrogels were affected by varying mass fractions of CHI‐CS, we combined PPP (40% w/v) in equal volumes with CHI‐CS (2, 3.5, and 5% w/v), designated the resulting hydrogels as CP2, CP3.5, and CP5.^[^ ^26^ ^]^ First, the morphology was observed by applying SEM, and the porosity was counted by ImageJ software. All hydrogels exhibited a characteristic 3D network structure, as shown in Figure 1E. As the concentration of CHI‐CS increased, the porosity decreased, and the structure became more uniform and denser (Figure 1F). Subsequently, the water absorption capability of the hydrogels was investigated by conducting swelling experiments. The absorption of all hydrogels in phosphate‐buffered saline (PBS) did not exceed their own mass, indicating good stability and the ability to maintain strength in moist environments. Additionally, the swelling rate of CP5 was slightly lower than those of CP2 and CP3.5, likely owing to its relatively high cross‐link density (Figure 1G). Next, we investigated the rheological properties of the CP hydrogels. As illustrated in Figure 1H,I, Figure S5a (Supporting Information), the energy storage modulus (G’) and loss modulus (G’’) of the hydrogels varied according to the temperature within the range of 25–50 °C. Specifically, the G’ and G’’ of the hydrogels increased with increasing temperature, and the structure of the hydrogels reached a critical state when the G’ and G’’ curves intersected.^[^ ^27^ ^]^ Beyond the intersection point, G’ remained higher than G’’, indicating that the hydrogel transitioned from the sol state to the gel state. The critical temperatures of CP2, CP3.5, and CP5 were 37.5, 37.3, and 33.7 °C, respectively. The change in gel temperature may be attributable to intermolecular hydrogen bond interactions between CHI‐CS and PPP, as well as covalent cross‐linking between catechol residues through self‐oxidation.^[^ ^17^, ^28^ ^]^ The maximum strengths of CP2, CP3.5, and CP5 at 37 °C were 265.2, 687.2, and 711.8 Pa, respectively, which is consistent with the cross‐link density results indicated by SEM. Additionally, the strength of the CP hydrogels increased with temperature, and the maximum strengths at 50 °C were 1975.3, 3188.1, and 8027.5 Pa, respectively. In summary, the strength of the CP hydrogels increased with increasing CHI‐CS concentration and temperature, with CP5 demonstrating enhanced rheological properties and higher strength. Based on these results, CP5 was selected for subsequent experiments.

Following the successful preparation of the CP hydrogels, BP@CP5 was obtained by further loading the BPNs. The addition of BPNs did not affect the temperature‐sensitive properties of CP5 (Figure 1D), but it decreased the porosity of BP@CP5 relative to CP5, leading to less swelling; this suggests that the incorporation of BPNs resulted in a denser network structure within the hydrogel, enhancing its stability in a wet oral environment. The EDS mapping results revealed that the P element was evenly distributed across BP@CP5 (Figure 1E), indicating a uniform distribution of BPNs throughout the CP5 hydrogel. DLS analysis confirmed the electroneutrality of PPP and that the zeta potentials of CHI‐CS, the BPNs, and BP@CP5 were 68.2, ‐32.5, and 30 mV, respectively (Figure S6, Supporting Information); this demonstrates that CHI‐CS can adsorb BPNs through charge interactions, thereby ensuring a homogeneous distribution of BPNs within the CP5 hydrogel. Notably, the surface charge of the main cariogenic bacteria was negative,^[^ ^11b^ ^]^ suggesting that BP@CP5 could effectively bind to cariogenic bacteria, thereby enhancing its antimicrobial efficiency. We also examined the rheological properties of BP@CP5. The gel temperature of the hydrogel decreased to 30 °C following the addition of BPNs, and the strength at 37 °C was ≈690 Pa (Figure 1I), which was not significantly different from that of CP5 (711.8 Pa); this indicates that although the inclusion of BPNs did not affect the strength of CP5, it reduced the gel‐forming temperature. Furthermore, the gel formation time was reduced to 60 s (Figure S4, Supporting Information), facilitating rapid gelling of the tooth surface to form a stable adhesive layer. These findings suggest that BP@CP5 not only retained the advantageous temperature‐sensitive and rheological characteristics of CP5, but it also possessed a denser network structure and enhanced stability in wet environments. Therefore, BP@CP5 is a promising candidate for effective antimicrobial and caries prevention applications in the oral cavity.

The injectability of hydrogels is crucial for their clinical application. Thus, shear‐thinning tests were conducted to determine whether the hydrogels were injectable.^[^ ^29^ ^]^ As illustrated in Figure 1J,K, Figure S5b (Supporting Information), all hydrogels demonstrated good injectability, and their apparent viscosity significantly decreased as the shear rate increased. Notably, both CP5 and BP@CP5 can be squeezed readily out of the tip of the needle, rapidly forming a stream of successive fine threads in the water and forming a “WMU” motif on a petri dish (Figure 1J,K, insert). Caries typically occur in hard‐to‐clean pits and fissures on the tooth surface,^[^ ^30^ ^]^ making it essential for caries‐preventive materials to possess a certain degree of fluidity to fully penetrate these areas. As illustrated in Figure 1L, we evaluated the fluidity of the hydrogel using a standard root canal preparation model,^[^ ^14c^ ^]^ finding that BP@CP5 could reach a depth of 5 mm in 60 s and penetrate the apices in 120 s; conversely, the varnish remained essentially immobile. Given that the average depth of the molar fossa is ≈1.5 mm,^[^ ^31^ ^]^ BP@CP5 can sufficiently penetrate the molar fossa to yield its photothermally active antimicrobial and enamel remineralization effects. These results highlight the excellent injectability and fluidity of BP@CP5, indicating its potential for effective use to prevent caries within the oral cavity.

### 2.2 In Vitro and in Vivo Wet Adhesion Properties

**Figure 2:**
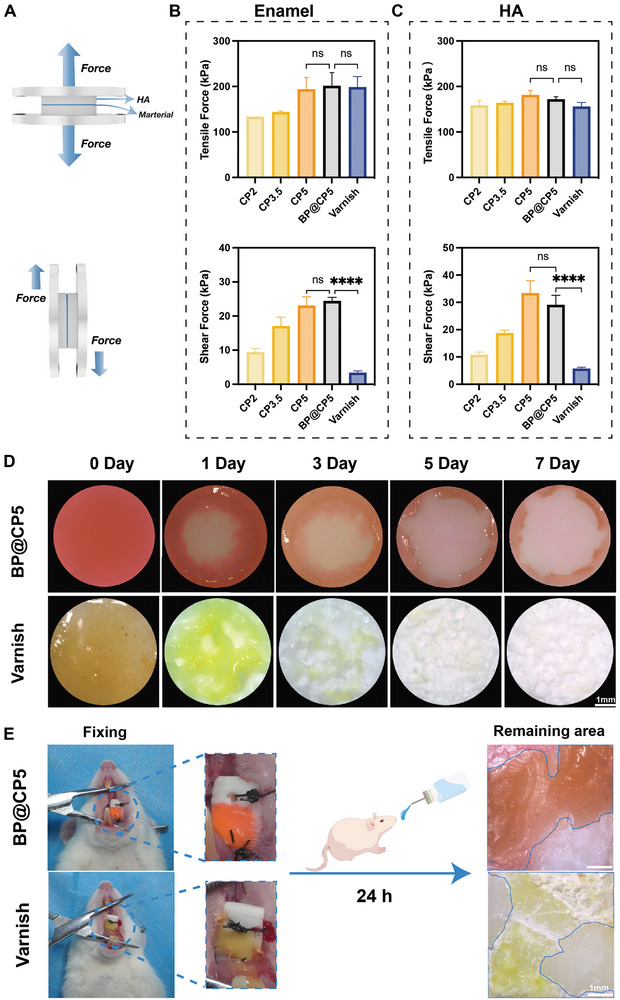
Wet adhesion properties of BP@CP5 hydrogel. A) Mechanism of wet adhesion. Tensile and shear adhesive force results for the varnish and various hydrogels on wet enamel (B) and HA (C) sheets. D) Long‐term wet adhesion properties of the varnish (fluorescein sodium staining) and BP@CP5 (rhodamine B staining) over 7 d. E) Wet adhesive properties of the varnish (fluorescein sodium staining) and BP@CP5 (rhodamine B staining) in vivo. In (B) and (C), the comparison is between BP@CP5 and CP5 or the varnish (*n* = 3), **** (*p* ≤ 0.0001), “ns” (Non‐significant).

The BP@CP5 hydrogel, based on mussel‐inspired chemistry, was designed to contain catechol groups that are known to form strong bonds with a variety of surfaces,^[^ ^17d^ ^]^ such as teeth, plastics, wood, glass, and rubber (Figure S7, Supporting Information). Given the dynamic and moist environment of the oral cavity, particularly the continuous saliva flow and oral movements, we evaluated the wet adhesion properties of BP@CP5. We selected varnish,^[^ ^32^ ^]^ an anticaries material with known adhesive capability, as a positive control, and we used an electronic universal testing machine (EUTM) to measure adhesion.^[^ ^15^ ^]^ **Figure** **2**A illustrates the experimental setup for testing the tensile and lap shear bond strengths. The tensile and shear adhesive strengths of the CP hydrogels increased with increasing concentration of CHI‐CS, as measured on enamel slabs (Figure 2B). This increase is attributed to the higher catechol group content in the hydrogel network, which promoted the formation of ionic bonds.^[^ ^17c^ ^]^ The tensile and shear adhesive strengths of BP@CP5 were not significantly different from those of CP5, indicating that the BPNs did not affect these properties. BP@CP5 reached tensile and shear adhesive strengths of ≈201.0 and 24.3 kPa, respectively. Notably, although the tensile adhesive strength of BP@CP5 was similar to that of the varnish (198.3 kPa), the lap shear bond strength of BP@CP5 was significantly higher than that of the varnish (3.3 kPa). We repeated the adhesion tests using standard hydroxyapatite (HA) sheets to confirm the reliability of the experiment and eliminate potential errors related to enamel slab preparation (Figure 2C). The results were consistent with the measurements on the enamel slabs (*n* = 3), further validating our findings. In summary, BP@CP5 was found to have excellent wet adhesion properties, making it a promising candidate for application in the dynamic and moist environments of the oral cavity.

To further simulate the conditions of intraoral temperature and saliva flow, we applied BP@CP5 and the varnish to HA sheets and immersed them in 6‐well plates filled with artificial saliva. The plates were then placed on a shaker table at 37 °C and 200 rpm; images were captured at specific intervals for observation (Figure 2D). After 24 h, the remaining content of each material exceeded 50%. By the seventh day, the remaining content of BP@CP5 was ≈31.4%, whereas that of the varnish was ≈42.81%; this indicates that the ability of BP@CP5 to maintain adhesion in a salivary environment is like that of the varnish, as no significant difference in adhesion was measured over a 24 h period.

The in vitro experiments demonstrated the excellent wet adhesion properties of BP@CP5. However, we conducted further experiments using a rat oral adhesion model owing to the influence of eating, oral activity, and other factors on hydrogel adhesion.^[^ ^33^ ^]^ As depicted in Figure 2E, enamel slabs were affixed to the buccal mucosa of the rats; BP@CP5 and the varnish were applied separately. The materials were removed after 24 h of normal water and food intake, and the remaining contents were calculated using ImageJ software. The results revealed that the remaining contents of BP@CP5 (53.52%) and the varnish (58.03%) were both above 50%, indicating that BP@CP5 maintained excellent wet adhesion performance in a complex oral environment. In conclusion, similar to the varnish, BP@CP5 demonstrated remarkable wet adhesion ability both in vivo and in vitro, laying a solid foundation for further clinical applications.

### 2.3 In Vitro Photothermal and PTT‐Driven Antibacterial Performance

**Figure 3:**
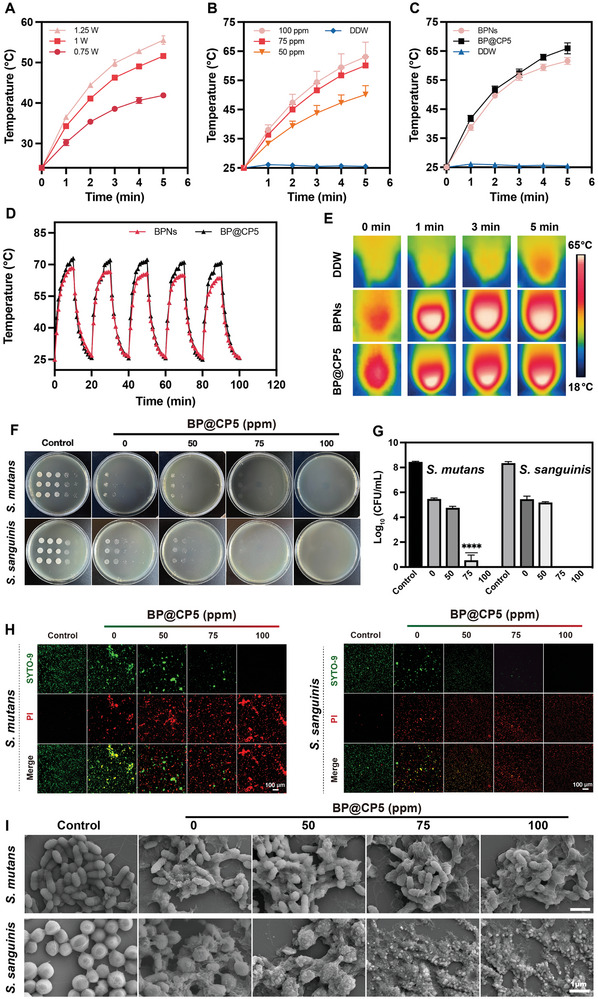
Photothermal and PTT‐driven antibacterial performance in vitro. Photothermal curves for the BPNs for different irradiation intensity (A) and different concentrations (B). C) Photothermal curves for the BPNs and BP@CP5. D) Photothermal stability of the BPNs and BP@CP5 for five on/off laser cycles. E) Thermal images of the BPNs and BP@CP5 at different durations of irradiation. Representative images of plate samples of *S. mutans* and *S. sanguinis* after being treated with BP@CP5 (different concentrations of BPNs; with 5 min of NIR) (F) and the number of surviving bacteria (G). Live/dead staining (H) and SEM images (I) of *S. mutans* and *S. sanguinis* after treated with BP@CP5 (different concentrations of BPNs; with 5 min of NIR). In (G), the comparison is between the control and BP@CP5 (75 ppm) (*n* = 3), **** (*p* ≤ 0.0001).

Considering that BPNs are key to the photothermal antimicrobial effect of BP@CP5,^[^ ^34^ ^]^ we initially explored the performance of concentration and radiation intensity on the photothermal characteristics of BPNs. As shown in **Figure** **3**A, the final temperature of the material increased with increasing radiation intensity, and significant heating was achieved at an irradiance of 1 W cm^−2^. Considering the adverse effects of excessively high radiation intensity on tissue health, we determined 1 W cm^−2^ to be the appropriate irradiance based on safety considerations.^[^ ^35^ ^]^ Figure 3B shows the results of subjecting different concentrations of BPNs and double‐distilled water (DDW) to 808‐nm near‐infrared radiation (NIR) at an irradiance of 1 W cm^−2^ for 5 min. The temperature of DDW remained largely unchanged, whereas temperature increases were observed for different concentrations of BPNs, with the 50, 75, and 100 ppm BPNs increasing by 25, 35, and 38 °C, respectively. This concentration‐dependent temperature increase confirms the photothermal activity of the BPNs. We examined the photothermal stability of BP@CP5 to assess the impact of hydrogel carriers on the photothermal activity of the BPNs. We subsequently further investigated the photothermal capability of BP@CP5. After 5 min of irradiation, the warming effect of BP@CP5 was stronger than that of BPNs alone, with an average temperature increase of ≈4.6 °C (Figure 3C); this demonstrates that the hydrogel effectively converts NIR radiation into thermal energy within a short period of time to reach sterilization temperatures. Generally, the photothermal stability and conversion efficiency (*η*) of a material critically affects its therapeutic efficacy.^[^ ^35^ ^]^ Using the cooling time illustrated in the cooling curves and ‐Ln(θ) (Figure S8, Supporting Information), the *η* values for BPNs and BP@CP5 were calculated to be 22.9% and 26.55%, respectively.^[^ ^36^ ^]^ This suggests that hydrogel loading enhanced the photothermal conversion efficiency of the BPNs. Furthermore, after five heating–cooling cycles, the highest temperatures of BP@CP5 and the BPNs decreased by 2 and 4.7 °C, respectively (Figure 3D); this indicates that the photothermal cycling stability of the BPNs was unaffected by hydrogel loading. Thus, BP@CP5 exhibited good photothermal stability and excellent photothermal conversion efficiency, making it a highly promising material for use in PTT‐based caries prevention.

Given that *Streptococcus mutans* (*S. mutans*) is the main cariogenic bacterium and *Streptococcus sanguinis* (*S. sanguinis*) is the first bacterium to colonize the tooth surface,^[^ ^37^ ^]^ we evaluated the antimicrobial activity of BP@CP5 against these two bacteria. Following treatment with BP@CP5 containing varying doses of BPNs, *S. mutans*, and *S. sanguinis* were isolated; their surviving bacterial populations were subsequently determined by counting the colony‐forming units (CFU) on brain heart infusion (BHI) agar plates (Figure 3F,G). Quantitative analysis revealed that the blank hydrogel exhibited antimicrobial activity, which has been attributed to the inherent cationic antimicrobial effect of CHI‐CS.^[^ ^38^ ^]^ The addition of BPNs endowed the system with a concentration‐dependent antimicrobial effect. The BP@CP5 with 75 ppm BPNs exhibited impressive bactericidal activity against *S. mutans* and *S. sanguinis*, their bacterial survival rates dramatically decreased from 8.4, 8.3 lg to 0.5, 0.0 lg, respectively. Live/dead staining, which was conducted using the isolated bacteria, revealed an antibacterial tendency similar to that of CFU counting (Figure 3H). We observed morphological changes in the bacteria by applying SEM to further investigate the antibacterial mechanism of BP@CP5. The blank hydrogel group exhibited bacterial cell membrane crumpling with no obvious structural disruption. Increasing the BPNs concentration and temperature was found to increasingly compromise the integrity of the bacterial cell membrane, leading to the outflow of bacterial contents. In the 75 ppm BPNs group, the cell membrane integrity of most *S. mutans* was lost, whereas the cell membranes of *S. sanguinis* were completely destroyed, with the bacterial morphology becoming indistinguishable. Remarkably, BP@CP5 enhanced the hydrophilicity of the tooth surface, facilitating the inhibition of bacterial attachment (Figure S9, Supporting Information).^[^ ^33^, ^39^ ^]^ Additionally, live/dead staining following BP@CP5 coating on cover slips and co‐cultivation with *S. mutans* resulted in a lower bacterial counts in the BP@CP5 group than in the PBS group, with a notable presence of dead bacteria (Figure S10, Supporting Information); this suggests that BP@CP5 has a strong antibacterial effect and some degree of biofilm formation inhibition.

### 2.4 In Vitro Degradation and Enamel Remineralization Performance

**Figure 4:**
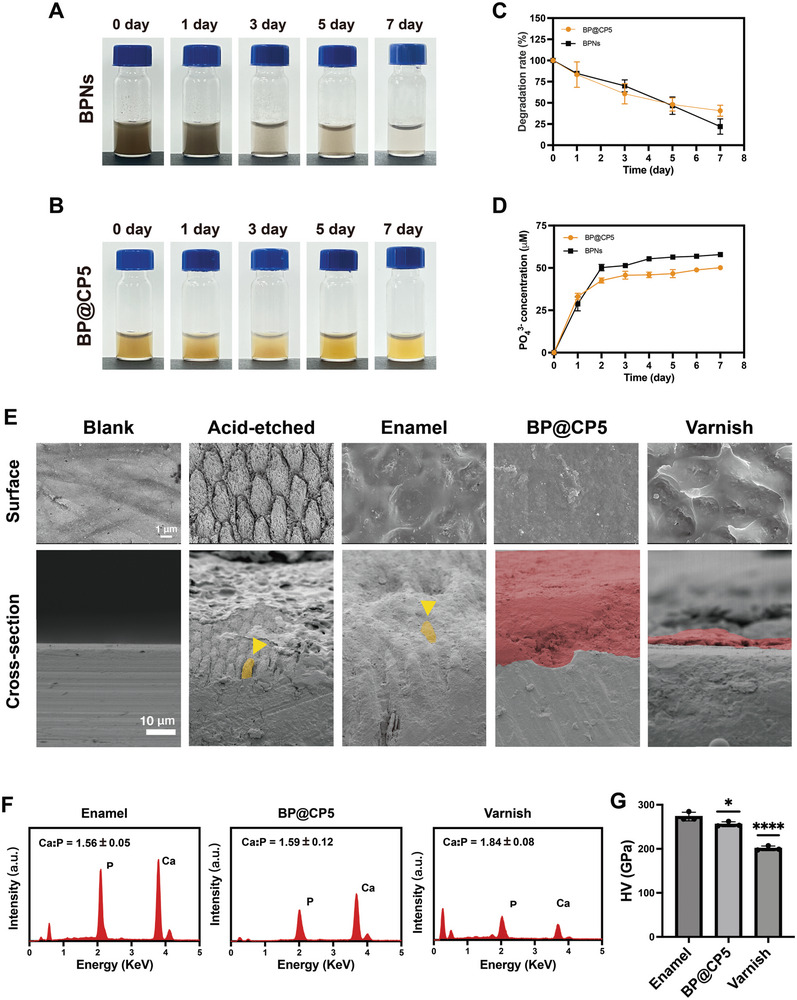
Degradation and remineralization performance of BP@CP5 in vitro. Images of the BPNs (A) and BP@CP5 (B) at Day 0, 1, 3, 5, and 7. C) Degradation behavior of the BPNs and BP@CP5. D) Phosphate generation capacity of the BPNs and BP@CP5. E) SEM images of the enamel slab and the corresponding cross‐sectional SEM images after various treatments. F) EDS spectra and Ca:P ratios for the natural enamel, BP@CP5, and varnish control. G) Surface hardness results. In (G), the comparison is between the enamel and other groups (*n* = 3), * (*p* < 0.05), **** (*p* ≤ 0.0001).

The degradation performance of the BPNs and BP@CP5 was further assessed by placing them in a horizontal shaker for 7 d. **Figure** **4**A illustrates the progressive fading of the BPNs, which lost most of their color and became transparent after 7 d, indicating time‐dependent degradation.^[^ ^14a^ ^]^ Figure 4B illustrates that the addition of BPNs consistently intensified the color of the CP hydrogel, suggesting a uniform dispersion of BPNs throughout the hydrogel. The Day 1, 3, 5, and 7 observations of diminishing BP@CP5 color indicated a gradual breakdown of the BPNs in the CP hydrogels. Its degradation products are nearly colorless, which also avoids the risk of tooth discoloration. Furthermore, UV–vis absorption curves (Figure 4C) and phosphate detection kits (Figure 4D) were used to investigate the degradation of BPNs in artificial saliva, as well as the concentration of PO_4_^3−^ ions in their post‐degradation solution. BPNs degraded by 30% within 48 h and 78% within 7 d. The concentration of PO_4_^3−^ in the solution steadily increased as the BPNs degraded, reaching 50.2 µm at 48 h and ≈58 µm on Day 7; this indicates the effective breakdown of BPNs in artificial saliva, with faster degradation in the initial 48 h and the continuous production of PO_4_^3−^, which is necessary for remineralization. To further verify the degradability of BP@CP5 and its ability to release PO_4_^3−^, the hydrogel was submerged in artificial saliva, periodically removed, freeze‐dried, weighed, and assessed for degradation. Approximately 39% of the hydrogel dissolved within 48 h, and after 7 d, only 40% remained intact (Figure 4C). BP@CP5 released a higher concentration of PO_4_^3−^ (≈43 µm) in the first 48 h, likely because of the faster degradation of the hydrogel during that period. Beyond 48 h, it continued to slowly degrade while continuously releasing PO_4_^3−^ (Figure 4D). In summary, BP@CP5 demonstrated good degradability in artificial saliva and effectively stimulated remineralization by producing a significant amount of PO_4_^3−^. Thus, this study provides a valuable reference for the design of in vivo experiments and their applications.

Building on these findings, we investigated enamel slabs to explore the remineralization properties of BP@CP5 in vitro. The remineralization results were assessed by applying Vickers hardness testing, SEM, and EDS.^[^ ^40^ ^]^ The SEM images (Figure 4E) revealed that the surface and cross‐section of the normal enamel had dense structures, whereas the acid‐etched enamel was observed to have exposed enamel rods with distinct crystal structures. In the blank group, mineralized material was visible on the enamel surface after 7 d of immersion in artificial saliva. However, the outlines of the enamel rods were still visible in the cross‐sectional image, indicating incomplete repair by saliva alone. Conversely, the BP@CP5 group was observed to have a dense coating of mineralized material on the enamel surface, with a newly formed mineralized layer of ≈15 µm that is visible in the cross‐sectional image. Although some mineralization deposits were observed on the enamel surface of the varnish group, there was no continuous layer of mineralization, and the surface was only partially mineralized; this demonstrates the significant remineralization capability of BP@CP5, particularly its ability to repair demineralized enamel. Analysis of the elemental composition (Figure 4F) revealed that BP@CP5 had high Ca and P contents, whereas the varnish group had relatively low Ca and P contents. In this experiment, the original enamel slab's Ca:P ratio was ≈1.56, which is consistent with previous reports on the Ca:P ratio of the normal enamel crystal structure (i.e., ≈1.67).^[^ ^41^ ^]^ Furthermore, the varnish group's Ca:P ratio was 1.85, whereas the BP@CP5 group's ratio was 1.60, which is closer to that of normal enamel; this further demonstrates that BP@CP5 effectively promotes remineralization, not only by repairing demineralized enamel but also by creating a crystal structure that is more similar to that of normal enamel. Additionally, the Vickers hardness test (Figure 4G) was applied to the newly formed enamel layers in each group.^[^ ^42^ ^]^ The hardness values for the original enamel, BP@CP5, and varnish groups were ≈273.32, 255.91, and 201.187 GPa, respectively. Despite being slightly lower than those for the original enamel, the BP@CP5 group's hardness values were much higher than those for the varnish group. In summary, BP@CP5 can help to remineralize demineralized enamel and repair enamel damage to some extent; additionally, the Ca:P ratio and hardness of the newly formed demineralized layer were closer to those of normal enamel than those of varnish.

### 2.5 In Vivo Dental Caries Prevention Effectiveness of BP@CP5

**Figure 5:**
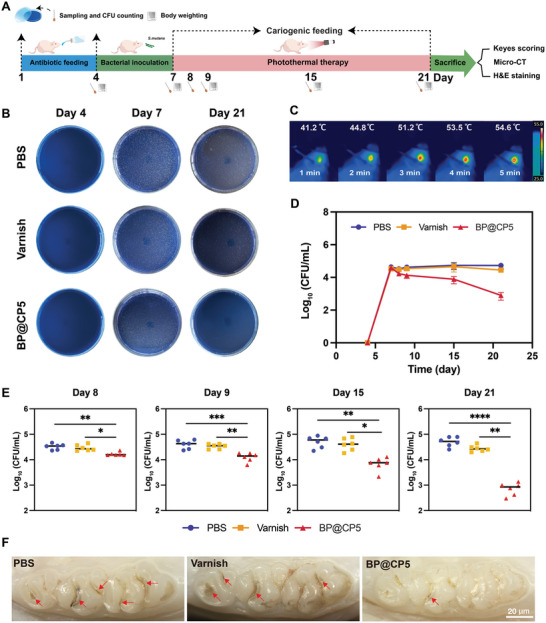
Prevention effect of BP@CP5 on dental caries in vivo. A) Animal Experiment Design Diagram. B) Representative images of surviving bacterial colonies on MSA plates for all three treatment groups at 4, 7, and 21 d (PBS; BP@CP5; varnish). C) Thermal images of the real‐time temperature of BP@CP5 in the rat molars after NIR irradiation. D) CFU counts for surviving bacterial colonies at Days 4, 7, 8, 9, 15, and 21. E) Results of statistical analysis for bacterial colonies that survived after BP@CP5‐mediated PTT. F) Representative photographs obtained by applying stereoscopic microscopy to the occlusal surface of treated rodent teeth on Day 21. In (E), the comparison is between PBS and the other groups (*n* = 6), * (*p* < 0.05), ** (*p* < 0.01), *** (*p* < 0.001), and **** (*p* ≤ 0.0001).

We established a rodent caries model using 6‐wk‐old, caries‐free male Sprague‐Dawley rats to further validate the effectiveness of the BP@CP5‐mediated PTT and antimicrobial and in situ remineralization capability in vivo.^[^ ^43^ ^]^ As illustrated in **Figure** **5**A, the experiment began by feeding the rats antibiotic‐supplemented food and water to eliminate endogenous *S. mutans*. This antibiotic feeding was conducted over Days 1–3. After confirming the clearance of endogenous *S. mutans* through oral sampling, exogenous *S. mutans* inoculation was initiated on Days 4–6, administered twice daily. Once the infection model was successfully established, BP@CP5‐mediated PTT was initiated once daily for two weeks starting on Day 7. Oral sampling and CFU counts were performed regularly throughout the caries modeling and PTT phases to monitor the number of cariogenic bacteria. At the end of treatment, the Keyes’ scoring system and micro‐CT were used to assess the depth and extent of caries in the different groups.^[^ ^43^, ^44^ ^]^ The rats were periodically weighed, and upon completion of the experiment, the oral mucosa and major organs were collected for hematoxylin and eosin (H&E) staining. Blood samples were collected for routine blood tests to evaluate the biosafety of the different treatments. This comprehensive approach ensured a thorough assessment of the antimicrobial efficacy and remineralization capability of BP@CP5, as well as yielded its safety profile in vivo. As shown in Figure 5B, no growth of *S. mutans* was observed on the Mannitol Salt Agar (MSA) medium for any group on Day 4, confirming that endogenous *S. mutans* was effectively inhibited. After 3 d of continuous inoculation with exogenous *S. mutans*, the growth of *S. mutans* was visible on the MSA medium for all groups, with the average number of bacteria in CFU count reaching ≈1 × 10^5^ CFU mL^−1^, demonstrating that the model of *S. mutans* infection was successfully established (Figure 5B,D). Subsequently, BP@CP5‐mediated PTT was performed. Figure 5C illustrates the real‐time temperature variation during the PTT, indicating that BP@CP5 largely maintained its photothermal capability in the animal's mouth. The temperature of the tooth surface after NIR irradiation for 5 min reached ≈54.6 °C, which is below the tolerance threshold for dentin and considered safe.^[^ ^11b^ ^]^ Figure 5D shows changes in the number of bacterial colonies during the treatment process for all groups. The curves for the PBS and varnish groups remained relatively stable, whereas the BP@CP5 group exhibited a decreasing trend in bacterial count. By Day 21, the number of bacteria in the PBS group slightly increased, which is consistent with caries development, whereas the varnish group demonstrated bactericidal efficacy of ≈19%. Conversely, over 97% of the bacteria were killed in the BP@CP5 group (Figure S11, Supporting Information), indicating a strong in vivo antibacterial effect of BP@CP5‐mediated PTT. The statistical analysis of the sampling results for each rat is illustrated in Figure 5E. Given the substantial antimicrobial effects of BP@CP5 in vivo, we hypothesized that it could also inhibit the progression of dental caries. Thus, using a stereoscopic microscope, the morphology and color of the maxillary molars of the rats were examined on Day 21 (Figure 5F). Most of the molars had carious lesions in the fossa, which are characteristic of early caries. Notably, more caries were present in the fossa of the PBS and varnish groups, whereas caries were observed in only a portion of the fossa in the BP@CP5 group (indicated by the red arrows). These findings demonstrate that BP@CP5 can effectively inhibit caries formation.

**Figure 6:**
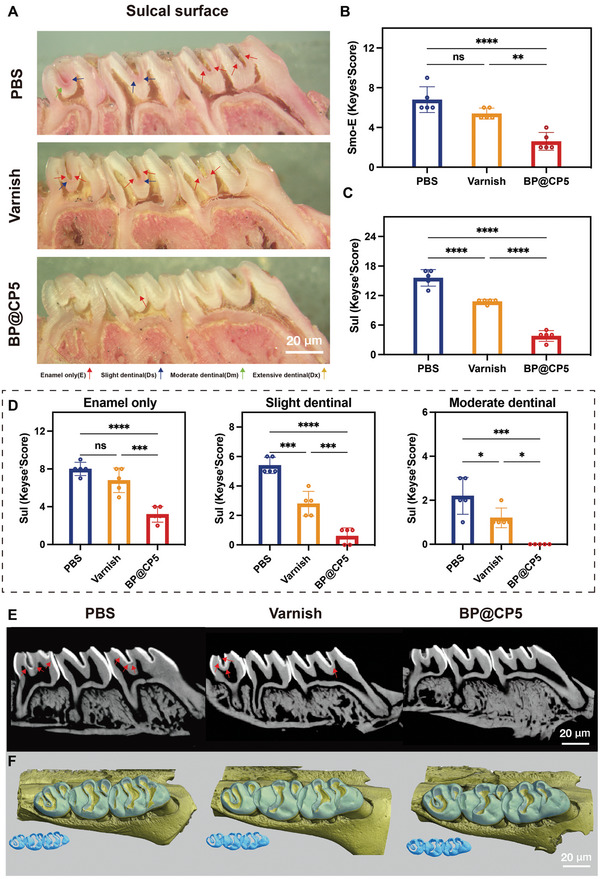
Anticaries effects after different treatments, as assessed via the Keyes’ scoring system and micro‐CT analysis. A) Representative images of carious lesions stained by murexide monohydrate on the sulcal surface of teeth with different severities (red arrows, affected enamel only, E; blue arrows, mild dentin, >25% of the dentin, Ds; green arrows, moderate dentin, 25–75% of the dentin, Dm; yellow arrows, extensive dentin, >75% of the dentin, Dx). B&C) Keyes’ scoring results for the smooth surface (B) and sulcal surface (C) based on the depth and extent of carious lesions (E + Ds + Dm + Dx). D) Keyes’ scoring results for the sulcal surface based on the depth and extent of carious lesions. E) Molars analyzed via micro‐CT analysis (red arrows, caries lesion sites). F) 3D reconstructions of micro‐CT images of maxillary molars for the three groups; enamel (blue) was distinguished by setting the density threshold above 4500 Hounsfield units. In (B), (C), and (D), the comparison is between each group (*n* = 5), * (*p* < 0.05), ** (*p* < 0.01), *** (*p* < 0.001), and **** (*p* ≤ 0.0001), “ns” (Non‐significant).

The Keyes’ scoring system and micro‐CT were employed to assess the depth and severity of caries in the different groups to further quantify the caries inhibition efficacy of BP@CP5. As illustrated in **Figure** **6**A, the area and depth of caries were marked by applying violet urate staining (red) as based on the Keyes’ score and categorized into four levels: enamel only (E, indicated by red arrows), mild dentin (Ds, <25% of the dentin area, indicated by blue arrows), moderate dentin (Dm, 25–75% of the dentin area, indicated by the green arrow), and extensive dentin (Dx, >75% of the dentin area, indicated by yellow arrows). The staining results show that the BP@CP5 group had a significantly better outcome in terms of the prevalence and severity of caries. The quantitative results show that the E scores on smooth surfaces were significantly lower for the BP@CP5 group (Figure 6B), whereas there was no significant difference between the varnish and PBS groups. Additionally, since early caries were mainly concentrated in the fossa, we quantified the severity of fossa caries damage and established four classifications: total damage (E + Ds + Dm + Dx), enamel caries (E), mild dentin damage (Ds), and moderate dentin damage (Dm) (Figure 6C,D). Total damage indicates the incidence of caries, whereas the other categories indicate the severity of caries. The results revealed that the varnish and BP@CP5 groups had lower incidences of caries than the PBS group and that BP@CP5 prevented caries more effectively than the varnish (Figure 6C). Furthermore, BP@CP5 effectively prevented caries progression and reduced its severity and prevalence (Figure 6D). Acid‐producing bacteria‐induced tooth demineralization is a crucial cause of dental caries formation, and and the extent of demineralization can be analyzed using micro‐CT.^[^ ^43^ ^]^ The corresponding sagittal section images show that the enamel of the BP@CP5 group exhibited reduced demineralization areas (Figure 6E, indicated by red arrows). In contrast, the PBS and varnish group fossae had demineralized sites that were larger in number and size, some of which even reached the pulpal cavity, indicating more severe damage to the dental tissues. Additionally, following the 3D reconstruction of the results for each group, the molar enamel was removed by applying a threshold to enable better visualization of the mineralization density, as shown in Figure 6F. High‐density intact enamel was present in the teeth of the BP@CP5 group (blue). These results suggest that BP@CP5 may be a powerful caries‐preventive agent owing to its efficient antimicrobial and in situ remineralization effects.

### 2.6 Biosafety Assessment

**Figure 7:**
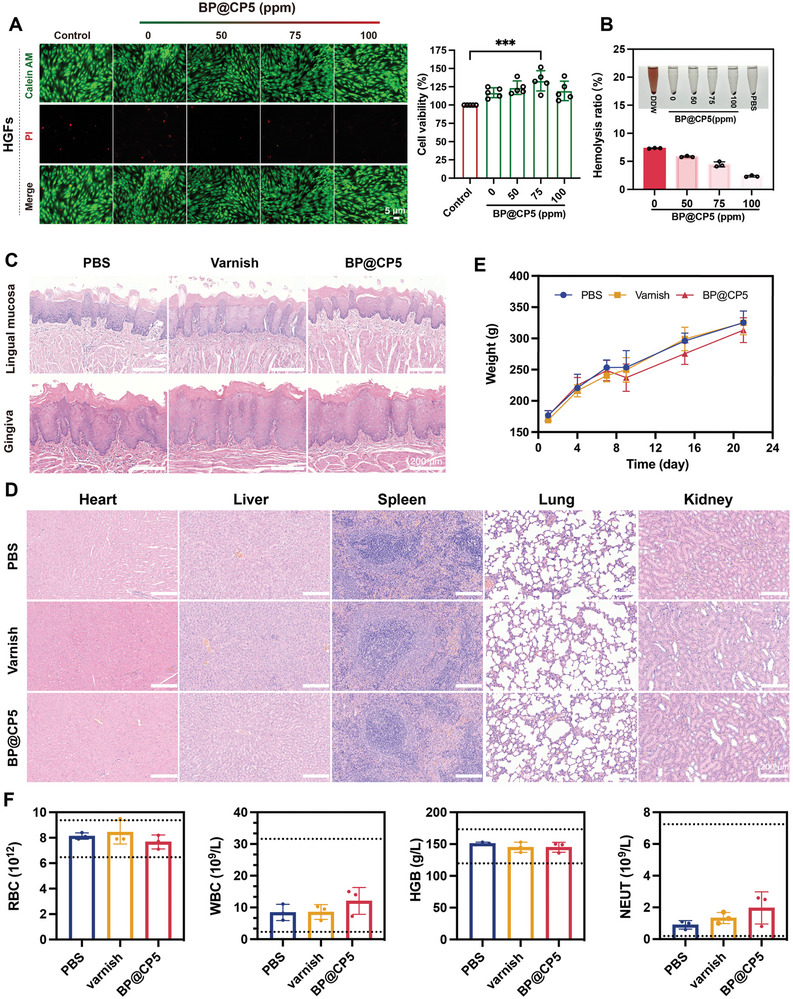
Biocompatibility of BP@CP5. A) Live/dead staining and cell viability of HGFs after various treatments over 24 h (*n* = 5). B) The hemolysis rate of BP@CP5 with varying concentrations of BPNs. C) H&E staining of oral mucosa and gingiva (C) and tissues of various organs (D) treated by the three systems. E) Body weights of rats subjected to one of the three treatments. F) Results of routine blood analysis for each treatment group on Day 21. In (A), the comparison is between the varnish control and BP@CP5 (75 ppm) (*n* = 5), *** (*p* < 0.001).

Biological safety is a prerequisite for the use of biomedical materials. First, we examined the in vitro safety of the hydrogels by applying a CCK‐8 assay, live/dead cell staining, and hemolysis experiments. Following a 24‐h incubation period with BP@CP5 (0–100 ppm) hydrogel extract, the viability of human gingival fibroblasts (HGFs) exceeded 100%, and that of mouse fibroblasts (L929) exceeded 90% for all experimental groups (**Figure** **7**A; Figure S12, Supporting Information). These results were consistent with the live/dead staining results. Furthermore, all samples had a hemolysis rate below 10% (Figure 7B). These findings collectively demonstrate the biosafety of the hydrogels in vitro.

To further investigate in vivo biosafety, we regularly weighed the rats and collected vital tissues and organs for H&E staining, as well as blood for routine blood analysis at the end of the treatment period. As illustrated in Figure 7C,H&E staining of the tongue and gingival tissues, which are the areas most likely to come into direct contact during caries treatment, revealed no significant tissue damage in any of the treated rat groups; this suggests that BP@CP5 did not damage the surrounding tissues during treatment. Additionally, there were no discernible differences between the groups in terms of body weight changes or H&E staining of the vital organs (Figure 7D,E). Furthermore, blood test indicators, including red blood cell count (RBC), hemoglobin (HGB), neutrophil count (NEUT), and white blood cell count (WBC), were all within the normal ranges for each group (Figure 7F). In summary, BP@CP5 demonstrated good biosafety, indicating its development as a safe and effective modality for caries control.

## 3 Conclusion

A multifunctional hydrogel platform (BP@CP5) with wet adhesion, antimicrobial, and in situ remineralization properties has been successfully developed. Benefiting from the photothermal activity and biodegradation products of BPNs, BP@CP5 exhibited highly efficient bactericidal and remineralization‐promoting effects. Specifically, when activated by NIR light, the high photothermal conversion efficiency of BPNs contributed to rapidly increasing the temperature, which eliminated cariogenic bacteria; moreover, the PO_4_^3−^ ions resulting from their degradation were able to bind to Ca^2+^ in the saliva to form calcium phosphate (CAP), which was confirmed to further promote the remineralization of the demineralized tooth surface. Furthermore, the loaded mussel‐inspired hydrogel ensures that the excellent effects of these BPNs can be realized in moist carious areas. Lastly, by applying BP@CP5 in a rat caries model, we found that it demonstrated excellent ability to prevent caries formation, as well as good biocompatibility. In summary, we have successfully developed a safe and effective drug candidate for the control of early caries.

## 4 Experimental Section

### Materials

Black phosphorus crystal powder (99.998%) was purchased from Zhongke Experimental Materials (China) and preserved in an inert atmosphere. The triblock PLGA_2K_‐PEG_1.5K_‐PLGA_2K_ copolymer was purchased from Shanghai Ponsure Biotech, Inc. The chitosan (CS, deacetylation degree ≥ 95%), N‐(3‐Dimethylaminopropyl)‐N′‐ethylcarbodiimide hydrochloride (EDC), 3,4‐Dihydroxyhydrocinnamic acid (HCA) was provided by Shanghai Aladdin Biochemical Technology Co., Ltd. (Shanghai, China). N‐Hydroxysuccinimide (NHS), Agar powder, phosphate buffered saline (PBS) and Brain Heart infusion (BHI) were obtained from Beijing Solebo Technology Co., Ltd. (Beijing, China). Mannitol Salt Agar (MSA) was provided by Qingdao Hope Bio‐Technology Co., Ltd. *Streptococcus mutans* (*S. mutans*, ATCC 25 175) and *Streptococcus S. sanguinis* (*S. sanguinis*, ATCC 12 598) were obtained from the American Type Culture Collection (ATCC, Manassas, VA, USA). Cell counting kit‐8 (CCK‐8), Calcein‐AM/PI cell viability assay kit, Malachite Green Phosphate Detection Kit were provided by Beyotime Biotechnology (Shanghai, China). HGFs was obtained from Otwo Biotech Inc (Shenzhen, China). The LIVE/DEAD baclight Bacterial Viability Kit was obtained from Thermo Fisher (Waltham, MA, USA).

### Synthesis and Characterization BPNs

BP Nanosheets (BPNs) were synthesized via liquid‐phase exfoliation,^[^ ^14a^ ^]^ beginning with black phosphorus in N‐methylpyrrolidone (NMP) (0.2 mg mL^−1^). Then the solution was sonicated using an ultrasonic cell pulverizer (Xiaomei XM150T, Jiangsu, China) for 1 h with a duty cycle of 3 s on and 2 s off, in an ice bath. This was followed by further sonication in an ultrasonic cleaner for 20 min over a total of 9 cycles. Centrifuge at 4000 rpm to remove the precipitate, then centrifuge the supernatant at 13 500 rpm, retaining the precipitate. Then washed with anhydrous ethanol, and the precipitate was resuspended. The final dispersion was stored at 4 °C in a sealed container. The zeta potential and particle size of the BPNs were determined using a Malvern particle sizer (Zetasizer Nano, UK). Their morphology was characterized by Scanning Electron Microscopy (SEM, Hitachi SU8010) and Transmission Electron Microscopy (TEM, JEM‐2100Plus).

### Catechol‐Modified CS

The EDC/NHS coupling reaction was utilized to couple catechol to the chitosan (CS) backbone.^[^ ^20b^ ^]^ Briefly, CS (504.2 mg) was fully melted in 5 mL (1 mol L^−1^) of HCL solution. Then, 49 µL of double‐distilled water (DDW) was added, and the pH was adjusted to 5. HCA (545.4 mg) was dissolved in 5 mL of RO water at pH 5 and added to the aforementioned solution. Concurrently, EDC (575.1 mg) and NHS (345.3 mg) were dissolved in a 50 mL ethanol/DDW mixture (1:1 v/v). The CS mixture was added dropwise with vigorous stirring at 25 °C for 10 h. The pH was maintained between 4.5 and 5.0. The final sample was purified by dialysis (MWCO: 3,500 Da) against HCl solution at pH 5.0 for 3 days and finally lyophilized for storage. The chemical characterization of CHI‐CS was studied by ^1^H NMR (AMX‐400, Bruker) and UV–vis absorption spectroscopy (Multiskan Go, Thermo Scientific). The degree of substitution of catechol was calculated by ^1^H NMR spectroscopy.

### Preparation of CP and BP@CP5 Hydrogel

CHI‐CS and PLGA‐PEG‐PLGA‐based blank hydrogels (hereafter referred to as CS and PPP, respectively) were made by blending equal volumes of PPP (40% w/v) and CS (2%, 3.5%, or 5% w/v). The hydrogels named as CP2, CP3.5, and CP5. The BP@CP5 hydrogel was prepared by incorporating ultrasonically stripped BPNs, adding lyophilized CS, and mixing it with the PPP hydrogel in equal volumes to form BP@CP5. The final thermosensitive hydrogel was composed of 20% PPP, 1.75% CS, and 75 ppm BPNs.

### Characterization of Hydrogel Properties

The sol–gel transition temperature as a function of time was determined using the test tube inversion method. This method was applied to measure the sol–gel transition temperatures of the PPP precursor solution, CP solution, and BP@CP5, using 10 mL reagent bottles. A system was considered to be in a state of gelation if no flow was seen over a 30 s period. The water absorption properties of the hydrogels were investigated through weight analysis.^[^ ^29^ ^]^ Aliquots of 2 mL of CP2, CP3.5, CP5, and BP@CP5 hydrogels were lyophilized (*n* = 3), weighed, and then added to 4 mL of artificial saliva. They were soaked until reaching solubility equilibrium at 37 °C. The hydrogel surface was blotted dry with filter paper, reweighed, and the swelling ratio was calculated. The equilibrium swelling ratio (ESR) was measured and calculated using the method reported, as described by Equation 1.^[^ ^45^ ^]^ In this equation, Wt denotes the weight of the swelling hydrogel and Wd denotes the weight of the drying hydrogel.

(1) $$\mathrm{ESR}\;=\frac{W\;t\;-W\;d}{W\;d\;}\;\times 100\%$$

The injection stability of the hydrogel was evaluated by observing its appearance when injected into water at 37 °C or extruded onto a receiving plate. If 1 mL of the hydrogel (labeled with sodium fluorescein) is loaded into a 26‐gauge syringe and then extruded directly through a needle, the hydrogel should remain in a continuous strip or form a “WMU” pattern in the water. Successful gelation and good stability are indicated if this occurs. The morphology of the hydrogels was analyzed with SEM, and porosity was computed with ImageJ software. The rheological properties of the hydrogels were evaluated using a HAAKE MARS rheometer (Thermo Mars40, USA).^[^ ^29^ ^]^ Shear thinning measurements were performed over a shear rate range of 0.1 to 1000 s^−1^ with strain and frequency held constant at 1% and 1 Hz, respectively; energy storage modulus (G’) and loss modulus (G’’) determinations were performed at a constant frequency of 1 rad s^−1^ over a temperature range of 20–50 °C (1 °C min^−1^ to cover the lower temperatures, room temperature, and body temperature); and at temperatures set to 25 and 37 °C cases to measure the effect of temperature on the viscosity of hydrogels.^[^ ^17c^ ^]^ The zeta potential of the hydrogels tested by Malvern Zetasizer Nano series (Zetasizernano ZS, UK). The flowability of the hydrogels was investigated using a root canal preparation model, in which appropriate amounts of BP@CP5 and Varnish were introduced into the model inlet, respectively, and the depths of penetration at 60, 120, and 180 s were observed at rest and photographed and recorded (*n* = 3), and the resulting images were measured using ImageJ.

### Wet Adhesion

First, prepared bovine enamel slabs were secured onto a base tray made of resin, and their surfaces were polished with 1000‐mesh sandpaper to achieve flatness, followed by acid‐etching for 30 s.^[^ ^40^, ^42^ ^]^ The enamel surface, except for a 5 mm × 5 mm square area, was covered with nail polish and then immersed in artificial saliva prior to use. Subsequently, 25 µL of each hydrogel group was applied to the surface of two enamel slabs. These were incubated for 5 min at 37 °C. The adhesive strength of the hydrogels was measured using an electronic universal testing machine (UTM 2102, Shenzhen Sun Technology Co., Ltd., China). The strength, expressed in kPa, was calculated as the maximum load in kN divided by the adhesive area in square meters (m^2^) (*n* = 3).^[^ ^15^ ^]^ The above experiment was repeated using hydroxyapatite (HA) tablets. Furthermore, to simulate the dynamic and humid oral environment, 10 µL of BP@CP5 (stained with rhodamine B) and 10 µL of varnish (stained with fluorescein sodium) were applied to the HA surface. The BP@CP5 group was irradiated with near‐infrared (NIR) light for 5 min, then immersed in artificial saliva and placed in a shaking incubator at 37 °C and 200 rpm. The samples were intermittently removed, rinsed under running water for 30 s, and photographed. To evaluate the in vivo adhesion effect of the hydrogels, pretreated enamel slabs were affixed to the buccal mucosa of rats. 10 µL of BP@CP5 (rhodamine B stained) and 10 µL of varnish (fluorescein sodium stained) were applied to the slabs. The BP@CP5 group was irradiated for 5 min (wavelength: 808 nm, intensity: 1.0 W cm^−2^). After 24 h, the enamel slabs were removed for microscopic observation of material adhesion.

### In Vitro Photothermal Effect

To assess the influence of concentration and radiation intensity on the photothermal characteristics of BPNs, solutions of BPNs (50, 75, and 100 ppm) were irradiated at a intensity of 1.0 W cm^−2^. Additionally, BPNs at a concentration of 75 ppm were irradiated at various intensities (0.75, 1.0, and 1.5 W cm^−2^) for 5 min and compared with DDW. Temperature changes were recorded for each minute using a thermal imager. To assess the impact of hydrogel carriers on the heating of BPNs, the temperature recorded after 5 min of NIR exposure to BP@CP5 (BPNs at 75 ppm) was compared with the results from the previous experiment.

### In Vitro Antimicrobial Activity

BP@CP5 containing varying doses of BPNs (0, 50, 75, 100 ppm) was used as the experimental material, with the irradiation time fixed at 5 min to investigate the concentration‐dependent antimicrobial activity of BPNs. Briefly, single colonies of *S. mutans* and *S. sanguinis* were cultured in BHI medium and incubated at 37 °C for 24  h, yielding a bacterial concentration of ≈1.6 × 108 CFU mL‐1. The bacterial precipitate was collected by centrifugation at 5000 rpm for 5 min and then resuspended in PBS solution. 100 µL of BP@CP5 was added to a 2 mL test tube, followed by the bacterial suspension (100 µL, 1 × 108 CFU mL^−1^), and the mixture was irradiated for 5 min using an 808 nm laser at a intensity of 1.0 W cm^−2^. Residual bacteria were collected, serially diluted, and homogeneously spread on BHI agar plates, then incubated at 37 °C for 24  h. The colonies were counted and the number of bacteria was indicated as log^10^ CFU mL^−1^. For the live/dead staining assay, the residual bacteria, acquired as described above, were first stained with 15 µL of PI (1.5 mm) and SYTO‐9 (1 mm) for 30 min, then put on slides and visualized using a fluorescence microscope (NI‐B, Nikon). Bacteria were fixed with 2.5% glutaraldehyde, dehydrated through an ethanol gradient, dried, and observed for morphology using SEM after gold sputtering. PBS‐treated bacteria served as controls.

### Degradation Performance

2 mL of BPNs solution was added to a 10 mL reagent bottle and placed in a shaker incubator set at 100 rpm and 37 °C for 7 days. The absorption spectra were measured at pre‐designed intervals of 0, 1, 3, 5, and 7 days. The concentration of BPNs was determined from the concentration‐absorbance standard curve, and photographs were taken at predetermined intervals. To track the degradation behavior of the hydrogel, BP@CP5 was first freeze‐dried and then immersed in DDW until a solubilization equilibrium was reached. Subsequently, the solubilized hydrogel was immersed in 5 mL of artificial saliva. The hydrogel samples were retrieved at regular intervals, rinsed with DDW three times to remove excess salts, and then freeze‐dried and weighed. The degradation rate of BP@CP5 was calculated using Equation 2, Where Wt is the current dry weight of the hydrogel, and Wi is the initial dry weight.

(2) $$\mathrm{Degradation}\;\mathrm{rate}\;\left(\%\right)=\frac{W\;i\;-\;W\;t\;}{W\;i\;}\;\times 100\%$$

In addition, phosphate ions (PO_4_^3−^) were detected using a Malachite Green Phosphate Detection Kit. Briefly, 200 µL of the solution to be tested (supernatant of BP@CP5) was mixed with 70 µL of the color developer, thoroughly mixed, and then left at room temperature for 30 min. The absorbance was measured at 630 nm using a spectrophotometer, and the phosphate concentration of the sample was calculated based on the standard curve and the dilution factor of the samples to be tested.

### In Vitro Enamel Remineralization

Healthy bovine incisors, free of caries and cracks, were selected and disinfected with 3% sodium hypochlorite, then rinsed with PBS. The labial enamel was cut to a thickness of ≈2 mm, and the labial dentin was similarly cut to a thickness of ≈2 mm, before being sliced into four separate pieces, each 1.5–2 mm thick. The slices were demineralized in Blue Etch acid etch for 60 s and then rinsed three times in an ultrasonic bath with DDW for 2 min each. The tooth sections were subsequently dried and stored at 4 °C before remineralization treatment. Enamel slabs were placed in 12‐well plates, with 2 mL of artificial saliva in each well. To these, 100 µL of BP@CP5, DDW, or Varnish were added. The experimental group was irradiated for 5 min and then placed in a constant temperature shaker incubator at 37 °C. Artificial saliva and medication were replenished every 24 h. After 7 days, the enamel slabs were removed to observe their morphology under a SEM, and the Ca:P ratio was assessed using X‐ray diffraction (XRD). The Vickers hardness of the slabs was measured using a Vickers hardness tester.

### In Vitro Biosafety Assessment

For live/dead staining assays, L929 or human gingival fibroblasts (HGFs) were first seeded into 96‐well plates (1×10^4^ cells per well) and incubated for 24 h. Subsequently, BP@CP5 extracts containing varying concentrations of BPNs (0, 50, 75, 100 µg mL^−1^) were added and the incubation continued for 24 h. The cells were rinsed with PBS, stained with calcein‐AM and PI (both at 5 µm), and examined using an inverted fluorescence microscope (IX70, Olympus, Japan). Regarding the CCK‐8 assay, cells were first rinsed with PBS and then exposed to 10 µL of CCK‐8 reagent for 0.5 h. Finally, the absorbance of each well was measured at 450 nm using a spectrophotometer. Cells treated with PBS served as controls. To test the hemolytic rate of the material, red blood cells (RBCs) were obtained by centrifuging fresh blood (1 mL) at 3000 rpm for 5 min, and then resuspended in PBS with a pH of 7.4. Hemolysate (50 µL) was added to the extracts (1 mL) prepared with different concentrations of BP@CP5, mixed by inverting, and incubated for 4 h at 37 °C. Afterward, the mixture was centrifuged at 3000 rpm for 15 min. The supernatant was photographed, and its OD_542 nm_ was measured and calculated using Equation 3.

(3) $$\mathrm{Hemolysis}\;\;\left(\%\right)=\frac{\mathrm{OD}\left(\mathrm{Sample}\right)-\mathrm{OD}\left(\mathrm{PBS}\right)}{\mathrm{OD}\left(\mathrm{DDW}\right)-\mathrm{OD}\left(\mathrm{PBS}\right)}\;\times 100\%$$

### Animal Experiment

All animal experiments were approved by the Wenzhou Medical University Laboratory Animal Ethics Committee, under the reference number wydw2023‐0662. Caries‐free rats were selected, given antibiotics for 3 days, and then infected with *S. mutans* (1 × 10^8^ CFU mL^−1^) for 3 days, with bacterial infection confirmed by plating.^[^ ^43^ ^]^ Rats were anesthetized with isoflurane and 2% sodium pentobarbital (0.2 mL 100 g^−1^). BP@CP5, PBS, and varnish were injected into maxillary teeth, followed by 5 min of 808 nm wavelength irradiation at 1.0 W cm^−^^2^ after BP@CP5 gel injection. Treatments were given daily from days 7 to 21, then continued daily to the experiment's end. Rats were water‐fasted for 30 min pre‐ and post‐treatment. Throughout the treatment period, they were fed a cariogenic diet (Keyes 2000) and given 5% sucrose water. Rat weights were consistently recorded alongside oral plaque sampling and CFU determinations. At the 21‐day mark, blood was drawn for standard analysis, and tissues including maxillary teeth, jaws, oral mucosa, and major organs were harvested. Caries severity was evaluated using Keyes' scoring and micro‐CT. The oral mucosa and various organs were subjected to H&E staining to assess the biosafety of the treatment.

### Statistical Analysis

Data are reported as mean ± SD from at least three trials. Statistical evaluations across all studies were conducted using one‐way analysis of variance (ANOVA). Data analysis was performed utilizing Prism software (version 10.0.3, GraphPad). Significance is marked as * (*p* < 0.05), ** (*p* < 0.01), *** (*p* < 0.001), and **** (*p* ≤ 0.0001). Non‐significant findings are labeled “ns”.

## Conflict of Interest

The authors declare no conflict of interest.

## Supporting information



Supporting Information

## Data Availability

The data that support the findings of this study are available from the corresponding author upon reasonable request.
