# Supplementary material for: Black Phosphorus Nanosheets‐Loaded Mussel‐Inspired Hydrogel with Wet Adhesion, Photothermal Antimicrobial, and In Situ Remineralization Capabilities for Caries Prevention
Source: Adv Sci (Weinh). 2024 Oct 11;11(45):2409155. doi: 10.1002/advs.202409155 (PMC11615761; doi:10.1002/advs.202409155)
Supplement: Supplementary file 1 — Supporting Information [file ADVS-11-2409155-s001.docx]

Supporting Information

**Black Phosphorus Nanosheets-Loaded Mussel-Inspired Hydrogel with Wet Adhesion, Photothermal Antimicrobial, and In Situ Remineralization Capabilities for Caries Prevention**

*Ying Ran, Jiayi Shi, Yiqin Ding, Lujian Li, Dandan Lu, Youyun Zeng, Dongchao Qiu,* *Jie Yu*, Xiaojun Cai*, Yihuai Pan**


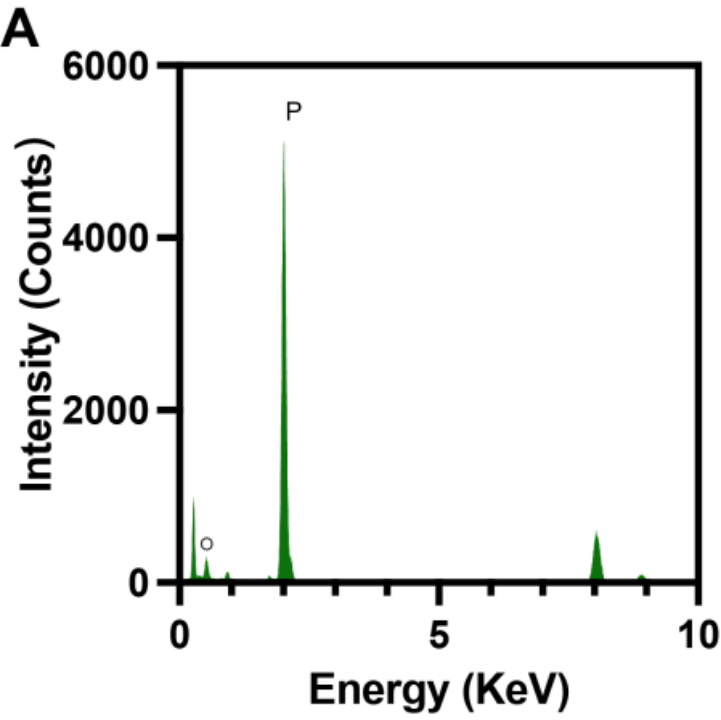


**Figure S1.** Distribution of elemental content in spectral sweeps of BPNs.**
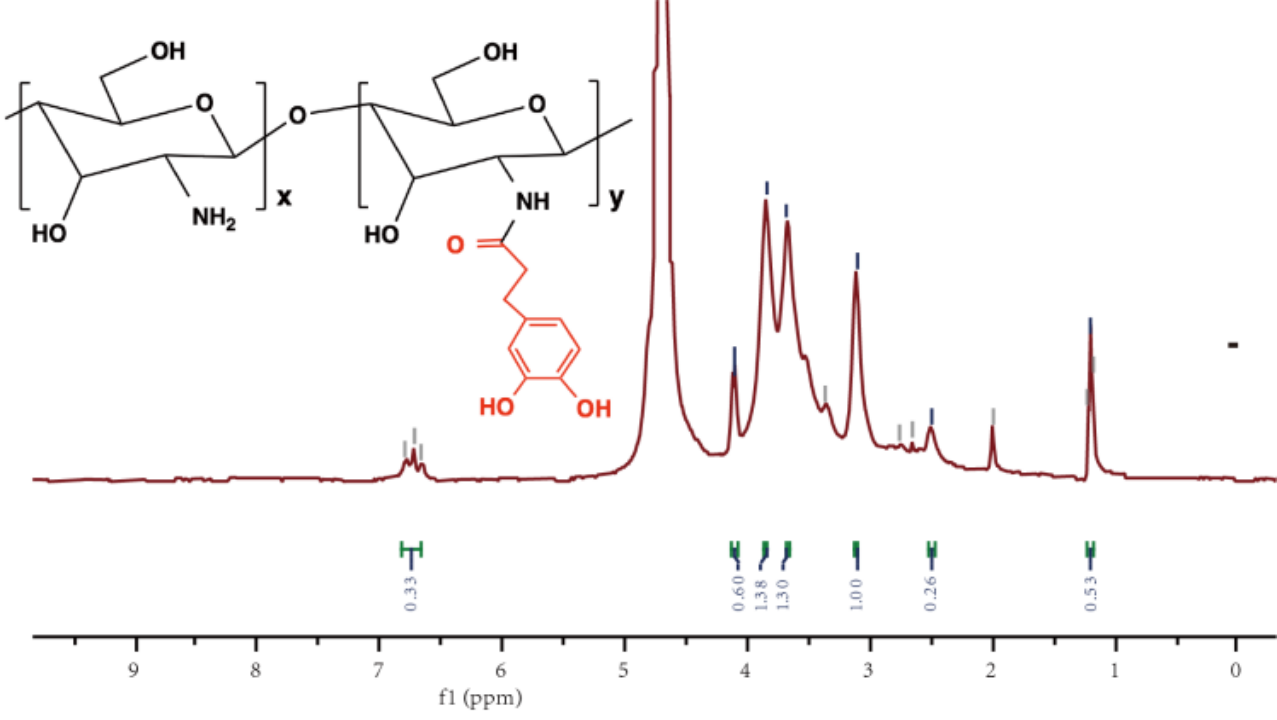
 Figure S2.** ^1^H NMR of CHI-CS

**
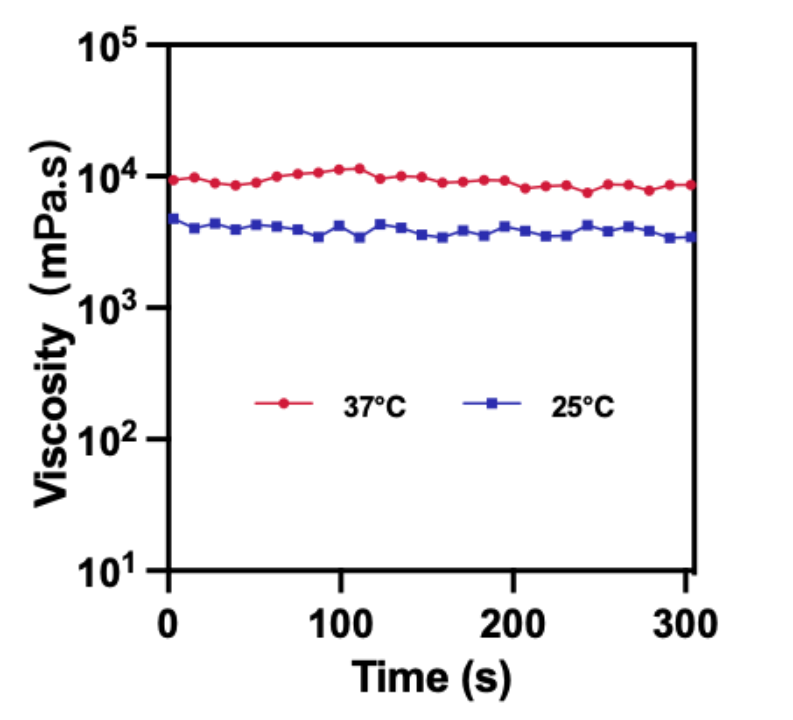
**

**Figure S3.** The viscosity of CP hydrogel at 25 °C and 37 °C.

**
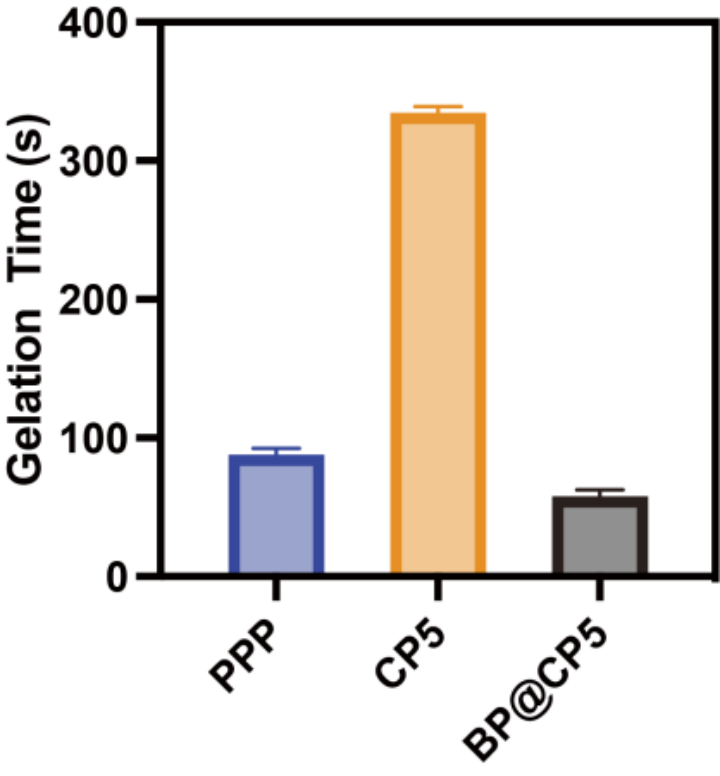
**

**Figure S4.** Gelation times of various hydrogels.


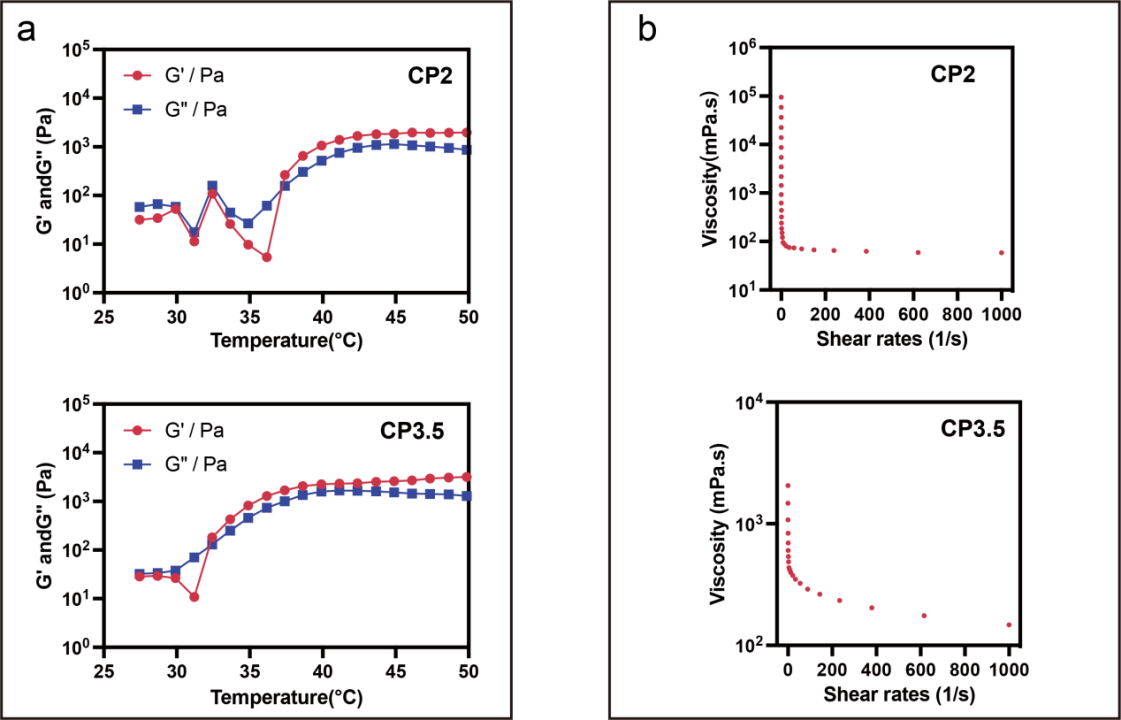


**Figure S5.** (a) Temperature-responsive storage (G’) and loss modulus (G’’) of CP hydrogel as a function of temperature from 25 to 50 ℃. (b) Shear viscosity.


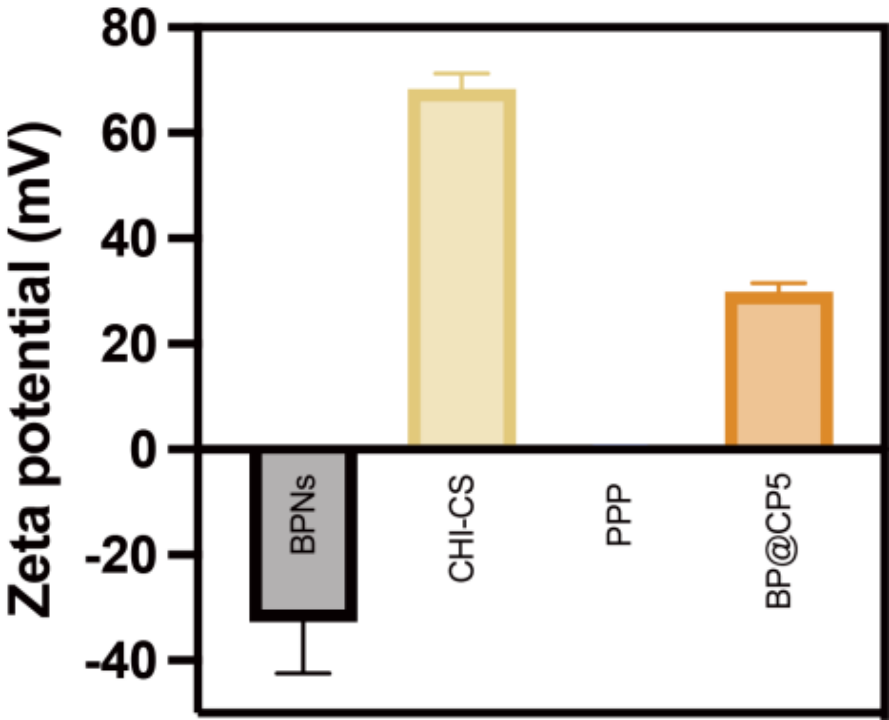


**Figure S6.** Zeta potentials of BPNs and various hydrogels.


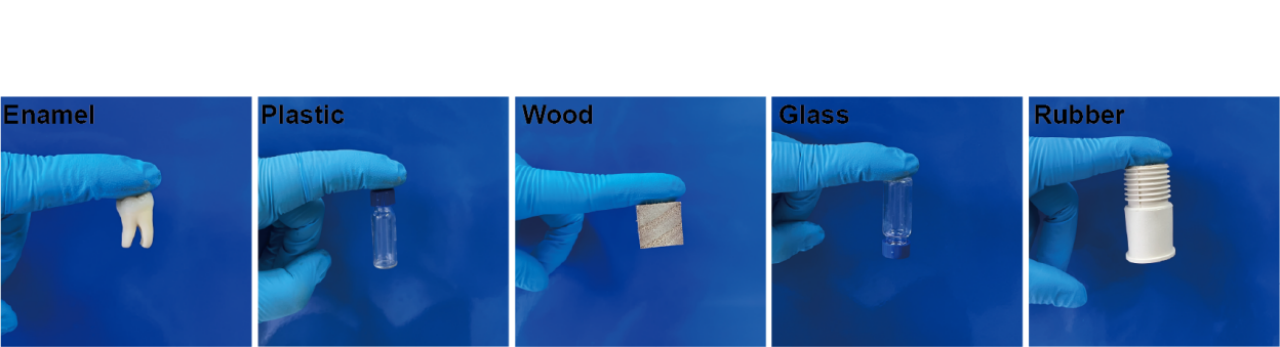


**Figure S7.** Photographs of BP@CP5 hydrogel adhering to different materials.


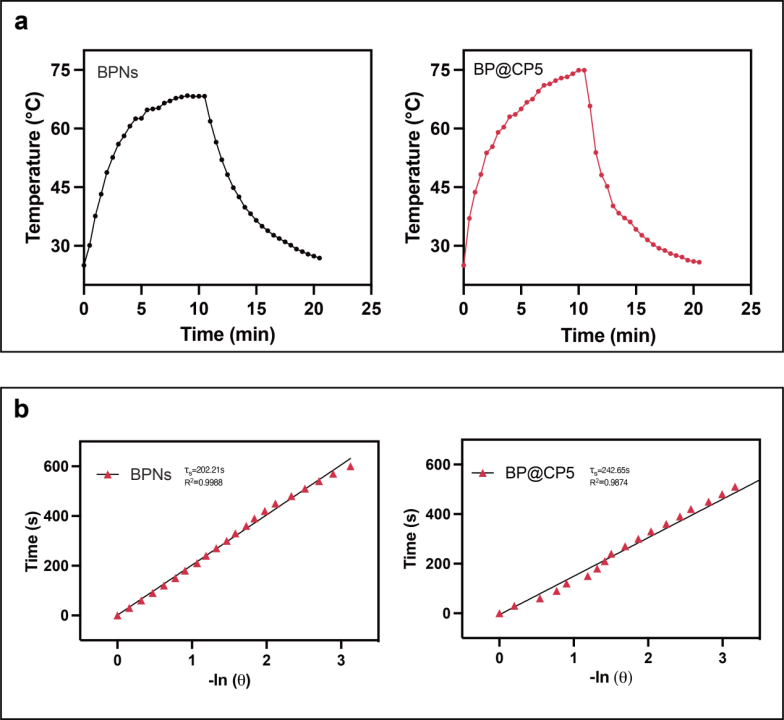


**Figure S8.** (a) Photothermal effect of BPNs aqueous dispersion and BP@CP5 hydrogel under irradiation of 808 nm laser with the power density of 1 W cm^−2^ and the laser was turned off after irradiation for 10 min. (b) The time constant for heat transfer from the system was determined to be τs by applying the linear time data from the cooling period (after 10 min) versus negative natural logarithm of the driving force temperature obtained from the cooling stage of (a).


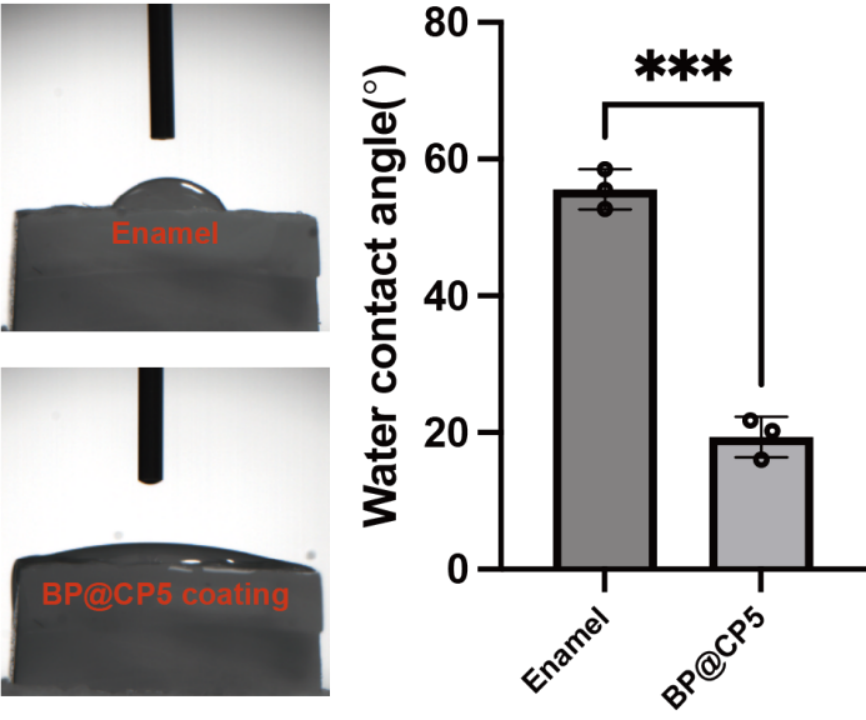


**Figure S9.** Water contact angle. (n=3)


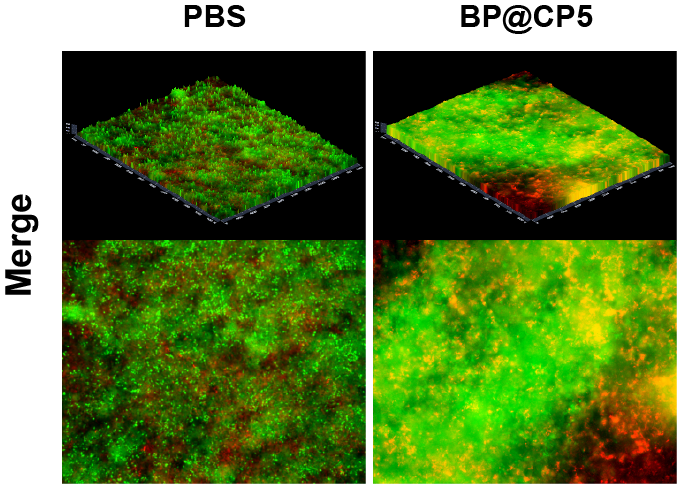


**Figure S10.** Live/dead staining of *S. mutans* after various treatments.


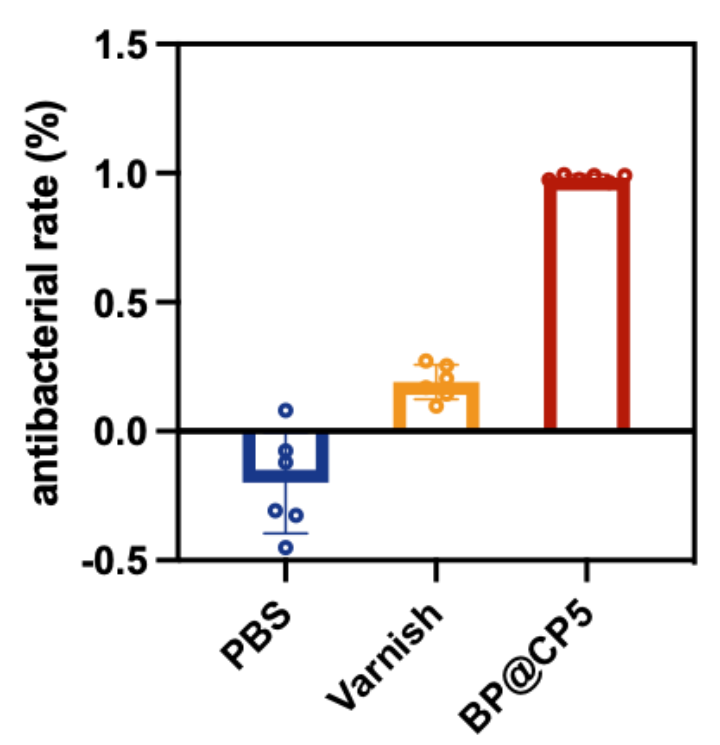


**Figure S11.** Statistical analysis of antibacterial rate after various treatments (n = 5).


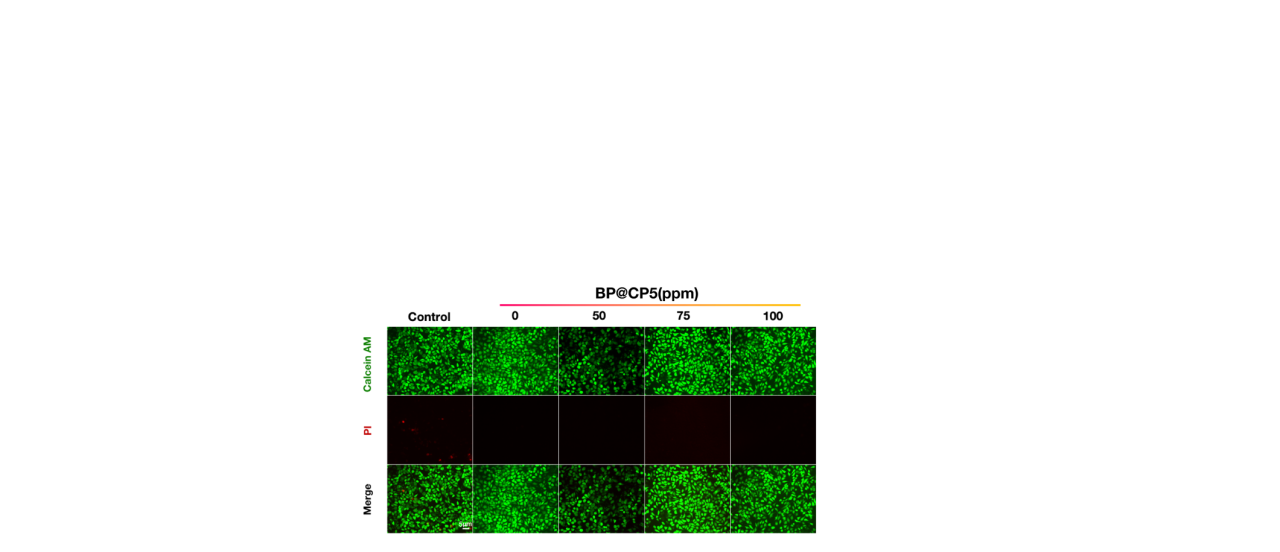

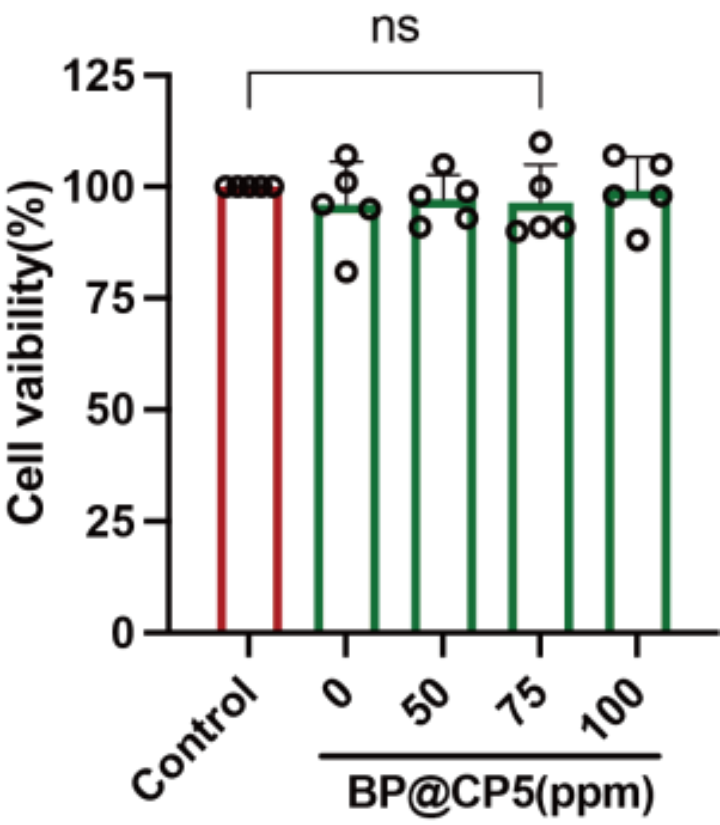


**Figure S12.** Live/dead staining and Cell viability of L929 after various treatments over 24 h (n = 5).

**
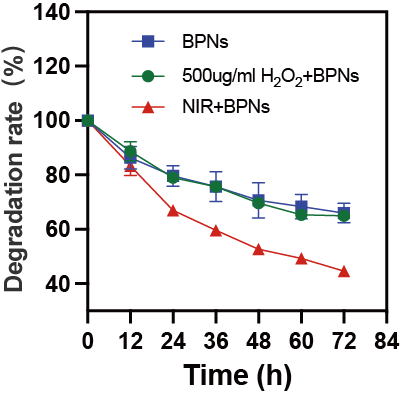
**

**Figure S13.** Factors affecting degradation (n = 3).
